# Advances in single-cell sequencing technology in the field of hepatocellular carcinoma

**DOI:** 10.3389/fgene.2022.996890

**Published:** 2022-10-11

**Authors:** Rongyi Qin, Haichao Zhao, Qizu He, Feng Li, Yanjun Li, Haoliang Zhao

**Affiliations:** ^1^ Third Hospital of Shanxi Medical University, Shanxi Bethune Hospital, Shanxi Academy of Medical Sciences, Tongji Shanxi Hospital, Taiyuan, China; ^2^ Shanxi Bethune Hospital, Shanxi Academy of Medical Sciences, Tongji Shanxi Hospital, Third Hospital of Shanxi Medical University, Taiyuan, China

**Keywords:** single cell sequencing, hepatocellular carcinoma, tumor heterogeneity, tumor immune microenvironment, circulating tumor cells, cancer stem cells

## Abstract

Tumors are a class of diseases characterized by altered genetic information and uncontrolled growth. Sequencing technology provide researchers with a better way to explore specific tumor pathogenesis. In recent years, single-cell sequencing technology has shone in tumor research, especially in the study of liver cancer, revealing phenomena that were unexplored by previous studies. Single-cell sequencing (SCS) is a technique for sequencing the cellular genome, transcriptome, epigenome, proteomics, or metabolomics after dissociation of tissues into single cells. Compared with traditional bulk sequencing, single-cell sequencing can dissect human tumors at single-cell resolution, finely delineate different cell types, and reveal the heterogeneity of tumor cells. In view of the diverse pathological types and complex pathogenesis of hepatocellular carcinoma (HCC), the study of the heterogeneity among tumor cells can help improve its clinical diagnosis, treatment and prognostic judgment. On this basis, SCS has revolutionized our understanding of tumor heterogeneity, tumor immune microenvironment, and clonal evolution of tumor cells. This review summarizes the basic process and development of single-cell sequencing technology and its increasing role in the field of hepatocellular carcinoma.

## 1 Introduction

Primary liver cancer (PLC) is one of the common gastrointestinal tract malignant tumors, and its morbidity and mortality have been high (Sung et al., 2021). LPC is histologically divided into three types: hepatocellular carcinoma (HCC), intrahepatic cholangiocarcinoma (ICC) and mixed hepatocellular carcinoma (cHCC-CCA). HCC accounts for more than 75% of PLC (McGlynn et al., 2015). China is a large country with high morbidity of liver cancer, which accounts for more than 50% of the total number of cases in the world (Zheng et al., 2018a). The current exploration of hepatocellular carcinoma has progressed from morphological studies in the early years to studies at the genetic level. As a disease closely associated with genetic material, tumors often show abnormal changes in cytogenetics and epigenetics, which are considered the major determinants of cancer at the both symptomatic and genetic levels (Lipinski et al., 2016). One of the long-standing challenges in biology and medicine is comprehensively characterize genotype-to-phenotype changes (Li and Clevers, 2010). However, the transcriptome information in any single cell reflects the activation of only a subset of genes, even though all cells in our body share nearly the same genotype (Huang, 2009). Furthermore, many different cell types in our bodies express unique transcriptomes, traditional bulk-sequencing are limited to measuring the average expression signal of cells contained in the tissue being tested (Shalek et al., 2014). A growing body of evidence further suggests that intracellular gene expression or transcriptome information is inconsistent even within similar cell types. However, at present, most transcriptome analysis are still based on the hypothesis that the genetic information of cells within the sampled tissue is consistent (Eldar and Elowitz, 2010). Thus, these researches may miss significant intercellular variability. For a better understanding of the biological processes of tumor progression, a more precise comprehension of the heterogeneity of genetic information in single cells is critical for elucidating their role in tissues and understanding how abnormal gene expression contributes to tumor progression.

Recent research has shown that there are a large number of gene mutations in the cellular genome of HCC, and the mutation pattern is related to its epidemiological background (Kan et al., 2013). Current studies suggest that the pathogenesis of HCC is related to a certain degree of aberrant activation of telomerase transcription, somatic mutation, and integration of hepatitis B virus (HBV) DNA. Critical pathways and key oncogenes are also involved in tumorigenesis. The intricate pathogenesis leads to differences in HCC pathological types and therapeutic susceptibility, resulting in significantly different prognoses between patients (Shibata and Aburatani, 2014). This inter-individual complexity of genome and pathology makes HCC highly heterogeneous and poses great challenges for personalized gene-based therapy of HCC patients.

## 2 Single-cell sequencing technology

Previous oncology studies have largely relied on sequencing results from large tissue samples consisting of millions of cells, therefore only focused on the average expression of specific transcripts within the tissue. This type of study is influenced by the expression level of each gene as well as the origin of different sample tissues. In recent years, single-cell sequencing based on next generation sequencing (NGS) has developed rapidly. The first single-cell mRNA sequencing analysis was performed in 2009 (Tang et al., 2009), the first single-cell DNA sequencing analysis in human cancerous cells was performed in 2011 (Navin et al., 2011), and the first single-cell exome sequencing analysis was performed in 2012 (Hou et al., 2012). Past research has suggested that single cells with the same phenotype were regarded as the basic functional unit of a tissue or organ, but complex heterogeneity between cells was observed by deep sequencing of single cells. Single-cell sequencing is currently most widely used for single-cell transcriptomic sequencing technology (single-cell RNA-seq, scRNA-seq), and has been widely used in research and some clinical settings (Hwang et al., 2018). Sample preparation, single-cell isolation, sequencing library construction, and data analysis are key steps in the entire single-cell sequencing workflow.

### 2.1 Sample preparation

Compared to conventional sequencing, a unique feature of single-cell sequencing is the requirement to obtain single cells in a great condition. To avoid the effects of hypoxia and ischemia on tissue and cellular status after separation from the organism, common single-cell sequencing techniques initially require the use of freshly isolated tissue for cell dissociation. However, in practice, due to the restrictions of the required condition or professional equipment, it may be arduous to process samples in real-time. In addition, processing multiple samples after collecting them at different times can lead to batch effects, which can interfere with sequencing results (Lafzi et al., 2018). The above-mentioned requirements for samples present certain challenges for single-cell studies.

Traditional tissue preservation methods, such as ultra-low temperature freezing (-80°C or liquid nitrogen storage), can lead to intracellular crystallization and rupture of cell membranes, which can affect subsequent single-cell sequencing. However, the integrity of RNA molecules from cell lines or primary tissues can be maintained for several months when stored at -80°C or in liquid nitrogen with cryoprotectants (dimethyl sulfoxide or serum-free cell cryopreservation medium) (Guillaumet-Adkins et al., 2017). Furthermore, combining methanol fixation with droplet-based single-cell approach, isolated cells can be stabilized for several weeks while guaranteeing the accurateness of later single-cell sequencing data (Alles et al., 2017). The sample preservation methods described above allow the location and timing of sampling to be independent of following processing procedures, enabling the successful implementation of complex experimental designs and expanding the range of samples that can be sequenced.

Additionally, in the clinic, many of the tissue samples are stored in Formalin-Fixed and Paraffin-Embedded (FFPE) blocks. For several reasons, including variations in fixation and tissue processing, FFPE samples may harbor greater variation in quality than frozen samples, and researchers usually do not have much control of those histologically collected samples (Gaffney et al., 2018). The above factors complicate the study of FFPE blocks at the molecular level, hindering their application in single-cell sequencing. However, due to its easy long-term storage characteristics, many precious clinical samples are stored in FFPE blocks. It is expected that researchers will find a suitable solution to increase the utilization of FFPE blocks in the field of single-cell sequencing.

### 2.2 Single-cell isolation

After more than a decade of development, the SCS sequencing process has been continuously updated and revised. The single-cell isolation technique is the first step in obtaining genetic information from a single cell, and its progress has greatly expanded the research in the field of single-cell sequencing. Among them, limiting dilution analysis (LDA) is the most simple but cumbersome method (Kalisky and Quake, 2011). Usually, only about one-third of the prepared well information can be obtained in the well plate when diluted to a concentration of 0.5 cells per well, which is inefficient. Capillary extraction of cells under microscopic manipulation is only used for embryology experiments or specific disease research, which is time-consuming and low-throughput (Brehm-Stecher and Johnson, 2004); Magnetic-positive cell sorting (MACS) or flow-activated cell sorting (FACS) has become the most common strategy for isolation of highly purified single cells (Julius et al., 1972). This method begins by labeling cells with monoclonal antibodies that recognize specific surface markers and classify different cell subpopulations. Alternatively, for unstained populations, negative selection can also be used to obtain specific cell populations. Laser capture microdissection (LCM) techniques use a laser system to separate cells from a solid sample assisted by a computer system and can preserve the original spatial information (Nichterwitz et al., 2016). However, the disadvantage is that there is no guarantee that the obtained sequencing information is from a single cell.

Microfluidics technology enables high-throughput sequencing research and has become the most widely used method for SCS research at this stage. It is popular for its low sample consumption, low analytical costs, and ability to achieve precise fluidic control (Whitesides, 2006). There are two main types of microfluidics technology, one is the microfluidic-based microplate (chip) technology. The commercial platform Fluidigm C1 is widely used to provide automated single-cell lysis, RNA extraction, and cDNA synthesis for up to 800 parallel cells on a single chip. Compared to microtube-based techniques, this platform provides lower errors and less deviations. However, its main disadvantages include the low number of cells captured (<1000) and the limitation of the size of the cells analyzed. Another widely used single-cell isolation technique is droplet-based microfluidics, which allows oil droplets to be monodispersed in a continuous aqueous phase (Thorsen et al., 2001; Utada et al., 2005). Compared to standard microfluidic-based chip technology, this system requires a smaller volume, enabling the manipulation and screening of thousands or even millions of cells at a much lower cost. The commercial Chromium system from 10× Genomics provides high-throughput analysis of the 3′ ends of single-cell RNA with high capture efficiency, making it the most widely used method for single-cell sequencing today. A summary of each single-cell isolation method is presented in Table 1.

**TABLE 1:** Single cell separation method.

Item	Pros	Disadvantages	References
Continuous dilution method	Simple operation and low cost	Low throughput, cellular attrition, difficult to filter target cells	Kalisky and Quake, (2011)
Manual pipetting aspiration	Simple operation and low cost	low throughput	Brehm-Stecher and Johnson, (2004)
Machine microscopic operation	Precise separation	Inefficiency, equipment dependence and mechanical damage	Guo et al. (2017)
MACS	High throughput with high accuracy	Prone to mechanical cell damage Injury, requires a large number of cells	Guo and Cairns, (2019)
FACS	High throughput with high accuracy	Prone to mechanical cell damage Injury, requires a large number of cells	Shapiro et al. (2013)
LCM	High accuracy and sustain sample space information	Low efficiency, high cost, susceptible to contamination by adjacent cells	Nichterwitz et al. (2016)
Microfluidic Platform	High throughput, high accuracy, mostly used in commercial platforms	Requires a max limit of cell size	Macosko et al. (2015)

MACS, magnetic-activated cell sorting; FACS, flow-activated cell sorting; LCM, laser capture microdissection.

### 2.3 Library construction

The steps required to construct a scRNA-seq library mainly include cell lysis, first-strand reverse transcription, second-strand synthesis, and cDNA amplification.

Cell lysis is usually performed in hypotonic buffer, followed by poly(A)+ selection using poly (dT) primers to capture messenger RNA (mRNA) in the cells. However, due to the principle of Poisson distribution, only 10%–20% of transcripts can be captured for reverse transcription at this step (Islam et al., 2014). Low mRNA capture-efficiency is a great challenge in the current scRNA-seq technology, requiring researchers to find and develop efficient cell lysis strategies.

cDNA preparation includes two parts: first-strand reverse transcription and second-strand synthesis. Currently, reverse transcriptases with low RNase H activity and high thermostability are often used for first-strand synthesis (Gerard et al., 2002; Arezi and Hogrefe, 2009). The second-strand is subsequently generated using poly(A) tailing (Sasagawa et al., 2013) or through a template switching mechanism (Islam et al., 2011; Ramsköld et al., 2012). Finally, a small amount of synthetic cDNA is further amplified using conventional PCR or *in vitro* transcription. The *in vitro* transcription method (CEL-Seq) can be used to amplify linear templates (Hashimshony et al., 2012), but the whole process is time-consuming and prone to 3′ coverage bias due to the need for additional reverse transcription (Jaitin et al., 2014). Smart-seq2 (an improved version of Smart-seq) generates full length transcripts and is therefore suitable for discovering variable splicing events and allele-specific expression using single nucleotide polymorphisms (Picelli et al., 2013). Currently, Illumina platforms (e.g., HiSeq4000 a n d NextSeq500) are widely used for sequencing steps. With the continuous improvement and maturity of library construction technology, the efficiency is greatly improved. In particular, the benchtop MiSeq sequencer has a fast library construction speed, which greatly reduces the turnaround time and can generate approximately 30 million paired-end reads in a single day.

Deep sequencing analysis often requires the detection of large numbers of cells. To reduce the sequencing cost, previous methods only focus on the 5′ or 3′ end of the transcripts (Islam et al., 2011; Hashimshony et al., 2012). More recently, researchers have incorporated unique molecular identifiers (UMIs) or barcodes (random 4–8 bp sequences) in the reverse transcription step (Islam et al., 2011; Hashimshony et al., 2012; Macosko et al., 2015). Considering the presence of 10^5^–10^6^ mRNA molecules and >10,000 expressed genes in a single cell, at least 4-bp UMI is required for sequencing library construction. UMI can help remove PCR duplicates and barcodes (random 4-8 bp sequences) can assign reads to their original cells. Using this strategy, the accuracy can be improved. However, current UMI tag-based methods only sequence the 5′ or 3′ ends of transcripts. Therefore, they are not suitable for the use of allele-specific expression. A comparison of representative scRNA-seq library construction methods is given in Table 2.

**TABLE 2:** Comparison of different scRNA SEQ techniques.

Approach	Sequencing range	UMI	Amplification method	Distinction	Bibliography
STRT-seq	5′ tag (TTS)	Yes	PCR	High precision, sequence-dependent bias	Islam et al. (2012)
Smart-seq/seq2	Overall length	None	PCR	High sensitivity, low efficiency	Ramsköld et al., 2012; Picelli et al., 2014
CEL- seq/seq2	Overall length	Yes	IVT	High precision, sequence-dependent bias	Hashimshony et al., 2012; Hashimshony et al., 2016
Quarz-seq/seq2	overall length	None	PCR	High sensitivity, high reproducibility	Sasagawa et al., 2013; Sasagawa et al., 2018
MARS-seq	3′ tag (UTR)	Yes	IVT	High sensitivity, high accuracy	Jaitin et al. (2014)
Drop-seq	3′ tag (UTR)	Yes	PCR	High efficiency, low cost, parallel analysis possible	Macosko et al. (2015)
inDROP-seq	3′ tag (UTR)	Yes	IVT	High throughput, low efficiency	Klein et al. (2015)
Cyto-seq	3′ tag (UTR)	Yes	PCR	Direct analysis of complex samples, which is relatively expensive and time-consuming	Fan et al. (2015)
Seq-well	3′ tag (UTR)	Yes	PCR	Portable, low cost, high throughput	Gierahn et al. (2017)
SCI-RNA-seq	3′ tag (UTR)	Yes	PCR	High-throughput, multi-round barcode combinations	Vitak et al. (2017)
SPLit-seq	3′ tag (UTR)	Yes	PCR	high efficiency	Rosenberg et al. (2018)

UMI, unique molecular identifiers; PCR, polymerase chain reaction; IVT, *in vitro* transcription.

### 2.4 Single-nucleus RNA sequencing

The main challenge faced in scRNA-seq is the complex experimental design. The tissue should be preserved intact or dissociated as single-cell suspension, fixed by methanol or formaldehyde. Cryopreserved or live cells and tissue should be dissociated using trypsin, cold-active protease, or a traditional method of digestion at 37°C (Aliya et al., 2022). The viability of different cell types in the process of tissue dissociation could introduce artifacts in cellular proportions or even completely lead to the loss of certain cell types. Artifacts of stress responses, such as heat-shock response, were observed in scRNAseq datasets that involved tissue dissociation. These all alter the transcriptional profiles of the cell types. Single-nucleus RNA sequencing (snRNA-seq) is an approach that bypasses the cell dissociation step required for scRNA-seq by using detergents to release nuclei from intact cells (Andrews et al., 2022). Enzyme-free isolation of the cellular nucleus could avoid the various potential problems described above. snRNA-seq sometimes enables the identification of rare subtypes that are indistinguishable by scRNA-seq (Andrews et al., 2022). However, T, B, and NK cells are underrepresented in snRNA-seq data and is therefore not recommended for studies in immune populations (Denisenko et al., 2020; Andrews et al., 2022). Overall, scRNAseq and snRNAseq could complement each other and help provide a more accurate full picture. A comparison of those two could be helpful in terms of understanding the results from different studies and provide guidance for selecting the best approach for one’s own study (Denisenko et al., 2020).

### 2.5 Single-cell DNA and epigenomics sequencing

In addition to the single-cell transcriptome, currently, the most widely used single-cell technologies are single-cell DNA sequencing (scDNA-seq) and single-cell epigenomics sequencing (scEpig-seq). A number of methods have been developed for single-cell DNA and epigenomics studies. Due to different amplification processes, the coverage, sensitivity, efficiency, and accuracy of these methods differ from each other. The characteristics and applications of the different methods are summarized in Table 3 and Table 4.

**TABLE 3:** Comparison of different scDNA-seq technologies.

Method	Scope	Features	Applications	References
DOP-PCR	Low	Exponential amplification, simple and fast operation, sequence-dependent bias, high allele loss rate	CNV	Arneson et al., 2008; Huang et al., 2015
MDA	Middle	Exponential amplification, High copy fidelity, Column-dependent bias, normalization invalid	SNV	Spits et al. (2006)
MALBAC	High	Linear amplification, high CNV detection accuracy, low false negative rate for SNV testing	CNV and SNV	Zong et al. (2012)
LIANTI	High	Exponential amplification, high genome coverage, high resolution of CNV	CNV	Chen et al. (2017)

DOP-PCR, degenerate oligonucleotide-primed polymerase chain reaction; MDA, multiple displacement amplification; MALBAC, multiple annealing and looping-based amplification cycles; LIANTI, linear amplification *via* transposon insertion; CNV, copy number variation; SNV, single nucleotide polymorphisms.

**TABLE 4:** Comparison of different scEpig-seq technologies.

Approach	Application	Features	Bibliography
scRRBS-seq	DNA methylation	Low throughput, low coverage	Guo et al. (2015)
scBS-seq	DNA methylation	Low throughput, low coverage	Smallwood et al. (2014)
scCGI-seq	DNA methylation	Low throughput, high coverage	Han et al. (2017)
scChIL-seq	Histone modification	Low throughput, high coverage	Harada et al. (2019)
scCUT&tag	Histone modification	High throughput, low coverage	Kaya-Okur et al. (2019)
scChIC-seq	Histone modification	Low throughput, low coverage	Ku et al. (2019)
scATAC-seq	Chromatin isomerization	High throughput, high coverage	Buenrostro et al. (2015)

Moreover, researchers are not limited to single-omics or single-aspect studies in tumor research, but integrate multiple omics into a single cell or link different aspects of the epigenome. For example, two multitopic approaches, sn-m3C-seq (Lee et al., 2019) and scMethyl-HiC (Li et al., 2019), have described methods to obtain linked chromatin conformation and methylation data from the same single cell, using bisulfite conversion of crosslinked genomic DNA. The combination of DNA and RNA sequencing of a single cell by DNA-mRNA sequencing (DR-seq) (Dey et al., 2015) or genome and transcriptome sequencing (G&T-seq) (Macaulay et al., 2015) can reveal genomic variation between individual cells, thus explaining changes in transcript levels. Techniques to analyze the epigenome and transcriptome of the same cell have been used to reveal the regulatory role of methylation and chromatin accessibility in gene expression (Angermueller et al., 2016; Hu et al., 2016). Furthermore, single-cell triomics sequencing (scTrio-seq) has been developed to simultaneously obtain information about the genome, DNA methylome and transcriptome from a single cell (Hou et al., 2016).

In addition, spatial information is lost due to disruption of tissue samples during single-cell sequencing, Single-cell spatial transcriptomics, which combines scRNA-seq with spatial transcriptomics (ST), can help solve this problem (Longo et al., 2021). It preserves the native structure and interactions of cells within a tissue, helps to profile RNA expression in their native site, deepening our understanding of disease pathogenesis (Ramachandran et al., 2020). Of course, besides the single-cell sequencing-based approach, multiplexed single-molecule FISH-based approaches in spatial transcriptomics, such as Multiplex Error Robust Fluorescent *In Situ* Hybridization (MERFISH) (Chen et al., 2015) and Sequential FISH+ (seqFISH+) (Eng et al., 2019), also provide important information at the single cell resolution while preserving the spatial information.

In recent years, research utilizing SCS technology have exploded due to its ability to process a large number of samples and identify unknown cell types compared to traditional sequencing techniques (Proserpio and Lönnberg, 2016). Furthermore, pseudo time analysis *via* SCS visualization can explore possible cellular origins and delineate cell lineage trajectories in detail (Pijuan-Sala et al., 2019), and SCS has been widely used to explore the mechanism of tumorigenesis and metastasis, thereby improving the diagnosis and treatment of cancer (Navin et al., 2011).

## 3 Single-cell sequencing analysis of normal liver

Single-cell sequencing was primarily used in embryology and developmental research in the early days. Some scholars have used scRNA-seq to measure the full-length transcriptome of thousands of mouse hepatocytes, and used single-molecule fluorescence *in situ* hybridization (smFISH) to label specifically expressed genes to infer the specific distribution coordinates of cells in the hepatic lobules. Finally, a high spatial resolution zonal map of liver genes was obtained and significant regional expression was found in approximately 50% of liver genes (Halpern et al., 2017). Liver is the immunologically privileged organ of the human body. Some scholars selected liver samples from liver transplant donors and conducted a study on liver-resident immune cells (LrIC) after screening by CD45^+^ cells, which revealed the distribution rules and specific expression genes of T/NK cells, B cells, monocytes/macrophages (Zhao et al., 2020). In 2018, Toronto Hospital mapped the transcriptional profile of the human liver microenvironment through the transcriptional profiles of 8,444 parenchymal and non-parenchymal cells isolated from fresh tissues from 5 human livers, and for the first time described distinct macrophage populations in the human liver and identified their unique functional pathways (MacParland et al., 2018). In addition, some scholars explained the specific mechanism of the liver in adapting the immune cell network to maintain the body’s immune barrier through SCS (Stamataki and Swadling, 2020). In 2021, a study summarizes, integrates, and analyzes five scRNA-seq datasets and provides an interactive Open Access online cell browser for easy access to gene expression data from 28 healthy human livers (Brancale and Vilarinho, 2021). It provides highly valuable information for further insight into the transcriptomic architecture and stability of human liver in physiological conditions. A systematic comparison of matched scRNA-seq and snRNA-seq has provided high-resolution maps of parenchymal cell populations in healthy human livers and detected rare subtypes of cholangiocytes and mesenchymal cells (Andrews et al., 2022). In studies of human liver development, scRNA-seq analysis of human fetal and adult livers has described the developmental trajectories of the different cell types that make up the human liver (Wesley et al., 2022). Analysis of the structure and spatial location of the normal liver contributes to the study of the pathophysiology of liver diseases, especially liver cancer.

## 4 Heterogeneity of liver cancer tumor cells

The existence of intratumor heterogeneity (ITH) was first described in the 1800s by the pathologist Rudolf Virchow, who observed microscopic inconsistencies in tumor cell morphology (Brown and Fee, 2006). Tumor heterogeneity, proposed by Fidler in 1977, is an important feature of malignancy (Fidler and Kripke, 1977). Tumor heterogeneity may be manifested by differences in clinicopathological type and degree of differentiation; Differences in the genome, transcriptome, and epigenome on a molecular basis, Differences in tumor cell aggressiveness, growth rate, and immune evasion in each patient; And differences in morbidity, mortality, and treatment sensitivity by country, ethnicity, race, gender, etc. All above factors lead to enormous challenges in the lengthy treatment process of tumors. HCC is one of the most heterogeneous tumors, and t traditional histology-based genome or transcriptome sequencing is difficult to elucidate the tumor’s genetic material alterations (Lu et al., 2016). Compared with traditional sequencing technologies, the most significant advantage of single-cell sequencing technology is the analysis of intercellular heterogeneity. We summarize the major single-cell hepatocellular carcinoma studies in recent years, as shown in Table 5.

**TABLE 5:** Overview of recent studies on the tumor microenvironment of hepatocellular carcinoma at the single-cell level.

Year	Study	Methods	Sample	Summary
2016	Hou et al. (2016)	scTrio-seq (scRNA-seq, CNV, DNA methylation)	HCC tumor tissue	Multi-omics HCC heterogeneity analysis. Two subpopulations of HCC cells were identified using a tri-omics approach of single cell transcriptome sequencing, CNV analysis, and DNA methylation sequencing
2017	Zheng et al. (2017)	RNA-seq, scRNA-seq (SMART-seq), TCR profiling	HCC Tumor tissue, paracancerous tissue, peripheral blood	Single cell analysis against HCC T cells. Different subpopulations of T cells can be detected in the tumor microenvironment. Trajectory analysis revealed a transition from naive T cells to depleted or cytotoxic T cells.TCR sequencing revealed the distribution of T cells in the tumor. *LAYN* as a suppressor marker gene for depleted CD8^+^ T cells and Treg expression
2017	Shi et al. (2017)	TCR-seq, WES, multiregional	HCC Tumor tissue, paracancerous tissue, peripheral blood	Multi-omics and immunologic library studies targeting tumor-infiltrating lymphocytes (TILs). Spatial heterogeneity of intratumoral T cell subpopulations exists. tIL diversity correlates with immune response, but not with mutational load
2018	Duan et al. (2018)	Single-cell genome sequencing	HBV-related HCC tissue	HCC single-cell genome analysis. Clonal evolution and HBV integration are early events in HCC development. The genome remains stable during tumor progression. Early intrahepatic spread of an initiating HCC clonal subpopulation leads to multifocal tumor formation
2018	Zheng et al. (2018b)	scRNA-seq (SMART-seq)	HCC Tissue tumor and HCC cell lines	HCC CSC Study. Cancer stem cells exhibit a high degree of heterogeneity. When analyzed by applying bulk sequencing data, the genetic signature produced by cancer stem cells can be found to correlate with patient survival
2019	Zhang et al. (2019a)	WES, RNA-seq, MS, Metabolomics, CyTOF, scRNA-seq (microwell seq) multiregional	HCC Tumor tissue, paracancerous tissue, peripheral blood	Multi-omics HCC heterogeneity analysis. The multi-omics study classified hepatocellular carcinoma into 3 subtypes: immune activation, immunodeficiency, and immunosuppression. Immunome and metabolome corresponded to subtypes more precisely than transcriptome and proteome. The study did not identify metabolomics-related targets, but their status correlated with immune cell subsets
2019	Ma et al. (2019)	scRNA-seq	HCC, iCCA Tumor tissue	Single-cell analysis reveals intra-tumor heterogeneity of tumor cells. The greater the transcriptome heterogeneity, the shorter the overall survival of patients. A link between hypoxia-dependent vascular endothelial growth factor (VEGF) expression in tumors and TME was identified
2019	Zhang et al. (2019b)	scRNA-seq (10xGenomics+Smart Seq)	HCC Tumor tissue, paracancerous tissue, liver lymph nodes, peripheral blood, ascites	Single cell transcriptome analysis for HCC CD45^+^ immune cells. SMART-seq is able to distinguish closely related cell subpopulations, while 10x Genomics can be used to analyze rare cell populations with low cell numbers. LAMP3^+^ DCs are mature DCs from the tumor to the local LN and play a major role in T-cell activation. Different macrophage subpopulations exist in tumors, among which tumor-associated macrophages (TAM) are closely associated with survival prognosis
2019	Ho et al. (2019)	scRNA-seq	Tumour tissue (PDTX model)	HCC CSC Study. A study of tumor heterogeneity and stemness-associated HCC subgroups in hepatocellular carcinoma. A rare CD24^+^CD44^+^ subpopulation of HCC with specific oncogenic features was identified in EPCAM^+^ tumor cells
2019	Duan et al. (2019)	Flow cytometry, RNA-seq	HCC Tumor tissue, paracancerous tissue, peripheral blood	Analysis against HCC MAIT cells. Intratumoral MAIT cells expressed an effector memory phenotype and they exhibited significantly elevated heterogeneous molecules (PD-1, TIM-3, CTLA-4) while secreted IFNγ and IL-17 were significantly reduced
2020	Losic et al. (2020)	RNA-seq, DNA-seq, scRNA-seq, TCR-Seq, SNP Array	HCC Tumor tissue, paracancerous tissue	Multi-omics HCC heterogeneity analysis. RNA-seq, DNA-seq, TCR-seq and SNP array data from multiple regions of liver cancer samples to map spatio-temporal interactions between cancer and immune cells
2020	Zheng et al. (2020)	CyTOF analysis, scRNA seq (10xGenomics), TCR profiling	HCC Tumor tissue (T), tumor margin (L), paracancerous tissue (N)	Single cell analysis for CD4^+^/CD8^+^ T cells in HCC. CD4/CD8 double-positive T cells were enriched at the tumor margin and co-expressed with PD-1/HLA-DR/ICOS/CD45RO; 11 CD4^+^/CD8^+^ T cell subsets with different cytotoxicity, depletion and activation scores were further described
2021	Sun et al. (2021a)	scRNA-seq, snRNA-seq	Primary and early recurrent HCC Tumor tissue, paracancerous tissue	Single-cell study of early recurrent hepatocellular carcinoma. Early recurrent hepatocellular carcinoma had reduced Tregs, increased dendritic cells, and increased intratumoral infiltration of CD8^+^ T cells. T cells in recurrent tumors overexpressed KLRB1 (CD161) and exhibited an innate immune-like hypo cytotoxic state. The number of these cells correlated with prognosis
2021	Ho et al. (2021)	scRNA-seq (10xGenomics)	HBV-related HCC tissue	Analysis of tumor heterogeneity and immune microenvironment in single-cell transcriptomic HCC. Tumor-associated macrophages were found to suppress T-cell infiltration and regulate the immunosuppressive environment through TIGIT-NECTIN2 interactions
2021	Dong et al. (2021)	SMART-seq 2	HCC Tumor tissue, paraneoplastic tissue	Single-cell studies of tumor cell heterogeneity in HCC. The authors identified heterogeneous sub clones of tumor cells in HCC tissues, including 5 HCC and 2 hepatocyte subpopulations, and found that MLX interacting protein-like (MLXIPL) was usually upregulated in HCC single cells and tissues and correlated with prognosis in patients with hepatocellular carcinoma
2021	Sun et al. (2021b)	SMART-seq2	Circulating tumor cells (CTC), HCC cell line	HCC CTC single-cell analysis. CTCs in the blood of HCC patients are associated with stress response, cell cycle and immune evasion. In addition, CCL5 expression in CTCs was found to be regulated by p38-MAX; CTCs recruit Tregs *via* CCL5 to facilitate immune escape and distant metastatic dissemination
2022	Guo et al. (2022)	scRNA-seq (10xGenomics), scDNA-seq	HCC Tumor tissue, paracancerous tissue	CNV evolutionary studies of single cell genomic and transcriptomic HCC. The study validated the biphasic copy number evolution model of HCC; ploidy-resolved scDNA-seq revealed a common clonal origin of diploid and polyploid aneuploid cells, suggesting that polyploid tumor cells arise from genome-wide doubling of diploid tumor cells

### 4.1 Genetic mutations drive hepatocellular carcinoma tumor cell heterogeneity

Currently, the most widely accepted genetic theory is that tumorigenesis arises from the accumulation of somatic mutations, that is, tumors are formed by abnormal evolutionary accumulation of somatic mutations (McGranahan and Swanton, 2017). Copy-number variant (CNV) at the single-cell level are able to distinguish malignant from non-malignant cells, enabling single-cell analysis that is not possible with bulk-seq. There is a high degree of genetic heterogeneity within and between different tumor cell subsets, and in some tumors there are significant differences between primary tumor cell subsets and metastatic tumor cell subsets (Park et al., 2010). Tumor cell CNVs caused by genomic instability are a major feature of early tumor heterogeneity. Some scholars have explored how CNVs help tumor cells evolve in HCC and revealed a new biphasic copy number evolution model for HCC(100). Single-cell genome sequencing revealed distinct patterns of HBV-related HCC clonal evolution, and found that specific HCC tumor cells could be of monoclonal or polyclonal origin; and found that polyclonal tumors had a typical fused multinodular morphology and are the class of tumors with the highest intratumoral heterogeneity (Duan et al., 2018).

Intratumoral heterogeneity (ITH) is the main reason for the failure of targeted therapy and immunotherapy in HCC. Perturbations and Darwinian evolution among tumor cell sub-clonal populations drive the continuous evolution of newly generated tumor cells on a pre-existing genetic background (Saunders et al., 2012). However, in addition to tumor cell own mutations (intrinsic drivers), some nongenetic factors (tumor microenvironment, TME) also significantly increase tumor cell variability, resulting in a complex and evolving tumor cell population. Some scholars integrated RNA-seq, DNA sequencing, TCR-seq, and SNP array data from multiple regions of liver cancer samples to map the spatiotemporal interactions between cancer and immune cells. How these interactions reflect intra-tumor heterogeneity was investigated by correlating regional neoepitopes and viral antigen load with regional adaptive immune responses. Researchers have found that correlating different region-specific expression, viral antigen load, and regional adaptive immune responses can reflect the causes of ITH(94). The above findings suggest that the intrinsic genetic variation of tumor cells is the main driving force of heterogeneity, and the extrinsic microenvironment acts together to promote the increase of tumor heterogeneity, which ultimately leads to the failure of targeted therapy and immunotherapy and tumor progression of HCC.

### 4.2 Hepatocellular carcinoma tumor spatial heterogeneity

The spatial heterogeneity of tumors is often thought to result from branched clonal evolution driven by random mutations that accumulate in different regions during solid tumor development (Davis et al., 2017). The spatial heterogeneity of tumors is reflected in that tumor cells at the tumor margin exhibit high invasive and metastatic characteristics, while cells within the tumor tissue maximize self-proliferation by promoting angiogenesis and increasing metabolism. Multi-omics fusion studies have found that the genome, transcriptome, proteome, and metabolome of HCC take the partial area in the microenvironment as the unit, and there is great heterogeneity in different areas (Guo et al., 2022).

Although single-cell transcriptomics can be finely divided into different cell types for analysis, the isolation of single cells destroys information about their spatial localization in natural tissues and their proximity to each other, which will lose the original spatial information and fail to reveal local networks of *in situ* intercellular communication. Recently, scRNA-seq combined with Spatial Transcriptomics (ST) has provided a new solution (Longo et al., 2021). The Fudan Zhongshan Cohort Study sequenced 97 paired samples using scRNA-seq and ST and found that the formation of metastatic microenvironment was mainly due to the immunosuppressive effect of MRC1+ CCL18^+^ M2-like macrophages, resulting in significant spatial reprogramming in the liver (Wu et al., 2022). Analysis of the spatial characteristics of the tumor microenvironment (TME) in primary liver cancer revealed that the envelope of some tumors affects the continuity of cell clusters at spatial locations within the tumor, resulting in tumor cell transcriptome diversity and suppression of immune cell infiltration in the microenvironment. And the study found that at the boundary between tumor and peripheral tissue, the bidirectional interaction of ligand-receptor help to maintain the intratumoral structure and the spatial distribution of PROM1^+^ and CD47^+^ CSCs; this process is associated with TME remodeling and tumor metastasis (Wu et al., 2021). The above studies suggest that HCC spatial heterogeneity significantly affects tumor progression, leading to regional imbalance of immune cells and consequently to remodeling of the tumor microenvironment in HCC.

### 4.3 Tumor stem cell and circulating tumor cell research

Local area spread or distant metastasis of tumor is the main reason for the poor prognosis of cancer patients. The formation and occurrence mechanism of tumor spread and metastasis have not been fully revealed, which greatly limits the possibility of prolonging the survival time of tumor patients. However, the discovery of circulating tumor cells (CTCs), which lead to distant dissemination of tumors, and cancer stem cells (CSCs), which are required to maintain tumor growth, has revealed a possible mechanism for the metastatic cascade response. For this reason, CSC and CTC are becoming a hot topic of research in the field of hepatocellular carcinoma. It has been found that the intratumoral molecular heterogeneity of HCC is partly attributable to the presence of CSCs, and that CSCs at the single-cell level are phenotypically, functionally, and transcriptomically heterogeneous (Zheng et al., 2018b). In addition, some scholars have found that CD24^+^/CD44^+^- EPCAM^+^ cell subsets have specific gene expression profiles, and these stemness-related cell subclones in HCC enable tumor cells to acquire great genetic richness, leading to the failure of HCC targeted therapy (Ho et al., 2019). In addition, single-cell analysis of CTCs from different sources in HCC patients found that chemokine CCL5 is an important mediator of CTC immune escape (Sun et al., 2021b). Overexpression of CCL5 in CTCs is transcriptionally regulated by p38-MAX signaling, which recruits Tregs to facilitate immune escape and metastatic seeding of CTCs (Sun et al., 2021b).

## 5 Immune microenvironment for liver cancer tumors

**FIGURE 1:**
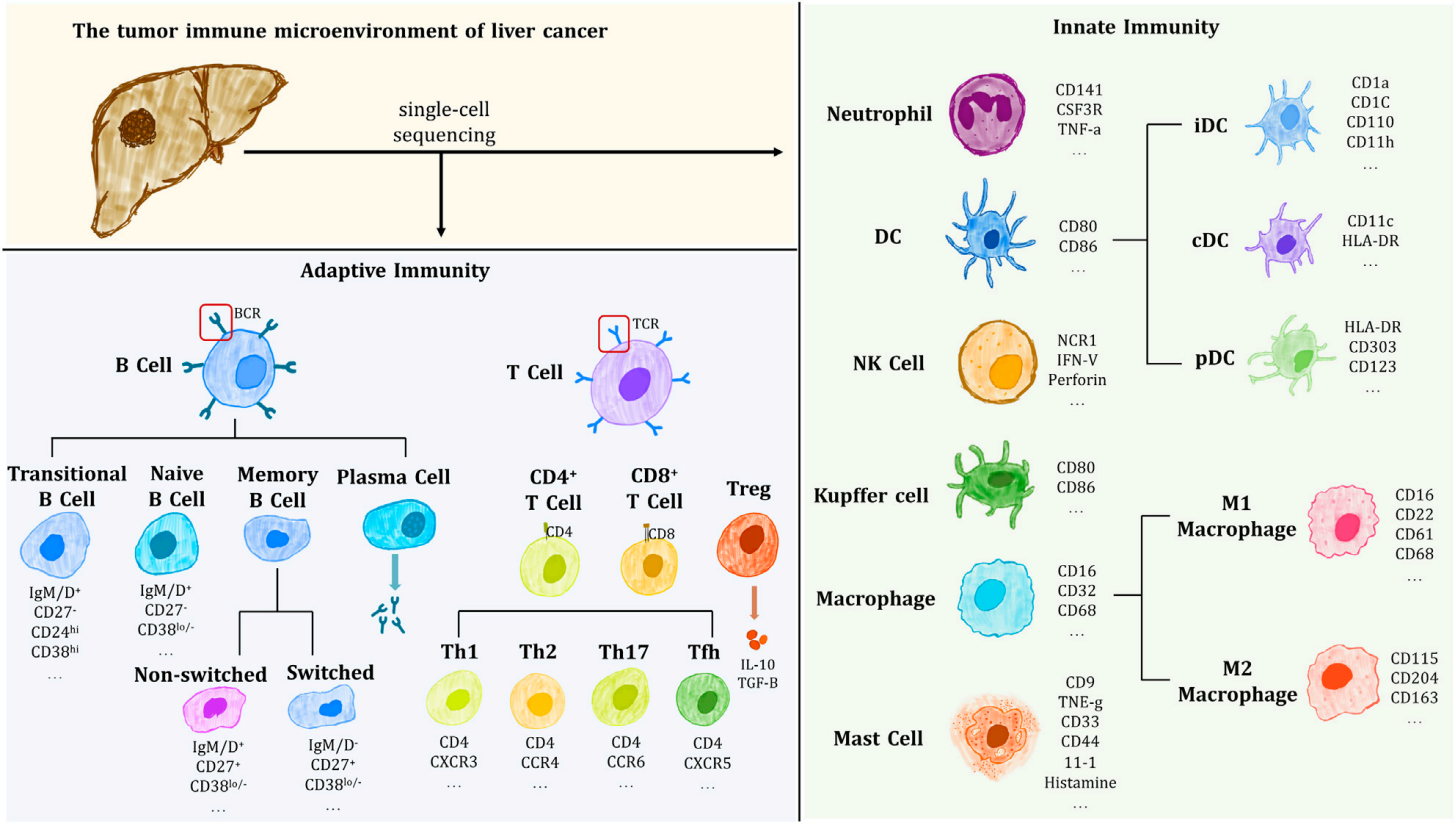
Immune cells and their markers isolated and identified in the immune microenvironment of HCC tumors.

Immune heterogeneity is an important aspect of tumor heterogeneity and is associated with drug resistance and immunotherapy. As shown in Figure 1, scRNA-seq confirmed that tumor tissue contains a complex immune component, including Innate immune cells such as dendritic cells (DC), immature dendritic cells (iDC), activated dendritic cells (aDC), eosinophils and neutrophils, mast cells, macrophages, natural killer cells (NK; NKCD56^-^ cells, NKCD56^+^ cells); Adaptive immune cells such as T helper cells Th1 and Th2, regulatory T cells (Treg), CD8^+^ T cells, central memory T cell (Tcm), memory effector T cell (Tem), T follicular helper cells (Tfh), γδ T cell, etc. The above-mentioned immune cells play their unique roles together in the microenvironment and constitute a complex and variable tumor immune microenvironment (TIME).

TIME plays a very important role in tumor heterogeneity and tumor progression (Wei et al., 2021). On the basis of the accumulation of random mutations in tumor cells during tumor development, there are differences in the immune effects of different regions of the tumor caused by TIME, and the evolution of tumor cell subclonal populations driven by this process is believed to be the main cause of the formation of spatial heterogeneity of HCC tumors (Lloyd et al., 2016). It is the selective role of the tumor microenvironment of liver cancer that leads to the continuous evolution of tumor cells toward the direction of optimal phenotypic characteristics, that is, the survival of a large number of well-adapted tumor cells (Lee et al., 2011). It has been proposed that the rate of tumor progression depends on a complex interaction between genetic and environmental factors (Wallace et al., 2015).

For the study of the immune microenvironment in HCC, SCS allows for more precise targeting of specific immune cell subpopulations. In 2017, The Center for Frontier Innovation in Biomedicine at Peking University selected T cells in HCC patients for deep single cell RNA sequencing, identified 11 T cell subsets based on their molecular and functional properties and delineated their developmental trajectories, and the genes characteristics of each subset were finally identified; And found that layilin was upregulated in activated CD8^+^ T cells and Tregs, and inhibited the function of CD8^+^ T cells *in vitro* (Zheng et al., 2017). In 2019, the same research team performed Single-cell sequencing of CD45^+^ immune cells selected from HCC tumors, liver lymph nodes (LN), blood and ascites, compared the two methods of SMART-seq and 10x Genomics, revealed that LAMP3^+^ DCs were mature DCs from tumor to local LN and play a major role in T-cell activation; different macrophage subpopulations exist in tumors, among which tumor-associated macrophages (TAM) are associated with the survival prognosis of HCC patients (Zhang et al., 2019b). Single-cell level studies have been conducted on HCC mucosa associated invariant T (MAIT) cells and found that MAIT cells were significantly enriched in the HCC microenvironment with upregulated expression of suppressor molecules such as PD-1, CTLA-4, and TIM-3, which correlated with poor clinical outcomes (Duan et al., 2019). Studies have found that TCR repertoires within a single tumor are low in similarity, and tumor-infiltrating lymphocyte (TIL) subsets differ between different regions of the same tumor; I Furthermore, correlation analysis showed that TIL diversity was significantly correlated with the expression of immunoreactive genes (Shi et al., 2017). According to the number and status of immune cells in the immune microenvironment, researchers divided HCC into three distinct HCC subtypes: immune-activated, immune-deficient, and immune-excluded (Zhang et al., 2019a).

In addition to acting on tumor cells, different immune cells also play an interactive role in TIME. Hong Kong scholars found that in HBV-HCC, TAM suppress tumor T-cell infiltration and regulate the immunosuppressive environment through TIGIT-NECTIN2 interactions (Ho et al., 2021). A single-cell study of early-stage recurrent HCC found that the number of Tregs decreased in recurrent foci, dendritic cells (DC) and intratumorally infiltrating CD8^+^ T cells increased; T cells in recurrent tumors overexpressed KLRB1(CD161) and exhibited a naive hypocytotoxic state (Sun et al., 2021a). Exploring the mechanisms of immune evasion associated with tumor recurrence provides deeper insights into the treatment of HCC tumor recurrence. In addition, some scholars sorted PD-1^high^CD4^+^CD8^+^ T cells by flow cytometry for single-cell sequencing, found the presence of 11 clustered CD4/CD8 double positive T cell (DPT) subpopulations with different cytotoxicity, depletion and activation scores, and which were enriched in the tumor marginal zone; The above DPT subsets are co-expressed with PD-1/HLA-DR/ICOS/CD45RO and affect the immune status of the microenvironment (Zheng et al., 2020). The above studies show that SCS technology (mainly scRNA-seq) can be used to discover the role of immune cells on tumor cells and the interaction between immune cells in TIME.

## 6 Outlook

In the era of molecularly targeted tumor therapy, the identification of predictive biomarkers is critical to the successful implementation of personalized medicine. However, tumor heterogeneity can be one of the major challenges of precision medicine. SCS can perform sequencing analysis and subtype classification of single cells, providing a favorable way to resolve tumor heterogeneity and better define immune microenvironment types, and hopefully become the theoretical basis for a new era of precision medicine. Overall, the prospects of SCS for tumor diagnosis, targeted therapy, and prognosis prediction are bright. In the near future, advances in SCS will undoubtedly improve our understanding of tumor biological characteristics, help us find potential therapeutic targets for patients, and then achieve precise tumor treatment.

## Data Availability

The datasets presented in this study can be found in online repositories. The names of the repository/repositories and accession number(s) can be found below: The transcriptome data are available in the Sequence Read Archive (https://www.ncbi.nlm.nih.gov/sra) at NCBI, with the BioProject ID: PRJNA859020 and SRA Accession Number: SAMN29766694-29766709.

## Glossary

PLC: primary liver cancer
HCC: hepatocellular carcinoma
ICC: intrahepatic cholangiocarcinoma
CSC: single-cell sequencing
HBV: hepatitis B virus
MACS: magnetic-activated cell sorting
FACS: flow-activated cell sorting
LCM: laser capture microdissection
scRNA-seq: single-cell RNA sequencing
UMI: unique molecular identifiers
PCR: polymerase chain reaction
IVT: *in vitro* transcription
scEpig-seq: Single-cell epigenomics sequencing
DOP-PCR: degenerate oligonucleotide–primed polymerase chain reaction
MDA: multiple displacement amplification
MALBAC: multiple annealing and looping-based amplification cycles
LIANTI: Linear Amplification *via* Transposon Insertion
CNV: copy number variation
SNV: singlenucleotide polymorphisms
DR-seq: DNA-mRNA sequencing)
G&T-seq: genome and transcriptome sequencing
scTrio-seq: single-cell triomics sequencing
LrIC: liver-resident immune cells
ITH: intra-tumor heterogeneity
ST: spatial Transcriptomics
TME: tumor microenvironment
CTCs: circulating tumor cells
CSCs: cancer stem cells
EMT: mesenchymal transition
DC: dendritic cells
iDC: immature dendritic cells
aDC: activated dendritic cells
Treg: regulatory T cell
Tcm: central memory T cell
Tem: memory effector T cell
Tfh: T follicular helper cell
TAMs: tumor-associated macrophages
VEGF: vascular endothelial growth factor
MLXIPL: MLX interacting protein-like
